# Preparation of Fluoro- and Bromofluoroaryl Compounds by Copyrolysis of Bromofluoroalkanes*

**DOI:** 10.6028/jres.065A.028

**Published:** 1961-06-01

**Authors:** Leo A. Wall, James E. Fearn, Walter J. Pummer, Robert E. Lowry

## Abstract

Pyrolysis of tribromofluoromethane yields chiefly hexafluorobenzene. Copyrolysis of this material with several bromine-containing compounds was studied at 540 °C and under several atmospheres’ pressure of nitrogen gas. The addition of bromine or dibromodifluoromethane has very little effect on the pyrolysis products of tribromofluoromethane. Copyrolysis with carbon tetrabromide or bromoform yields increased amounts of bromopentafluorobenzene and dibromotetrafluorobenzene at the expense of hexafluorobenzene. The addition of relatively small amounts of 1,1,1-tribromo-2,2,2-trifluoroethane gives a significant yield of octafluorotoluene.

## 1. Introduction

Synthesis of hexafluorobenzene by the pyrolysis of tribromofluoromethane has been described and investigated by several workers [1–4]^1^. The original synthesis [1] was carried out at atmospheric pressure and 640 °C. In a previous study of this reaction in our laboratory [2], we explored the effects of pressure and temperature. On increasing the pressure to 4 atm, optimum yields were obtained near 540 °C, and the maximum yields were somewhat greater than at atmospheric pressure. A secondary product from the pyrolysis of tribromofluoromethane was bromopentafluorobenzene[2,3]. Since this material has great value in synthesis work [5], it was of interest to find means of increasing the relative yield of this product. A conceivable approach to this aim would be to increase the concentration of bromine in the reaction or to introduce carbon tetrabromide or bromoform into the reaction. Introduction of other compounds, it was anticipated, could also lead to the synthesis of various derivatives of hexafluorobenzene or related compounds.

## 2. Materials

The tribromofluoromethane, obtained from Columbia Organic Chemicals, Inc., was dried over anhydrous calcium sulfate and filtered through glass wool. The carbon tetrabromide, bromoform, and bromine were reagent grades and were used without further purification. The 1,1,1-trifluoroethane was a research sample.^2^ The dibromodifluoromethane was obtained as a byproduct in the pyrolytic preparation of hexafluorobenzene.

The tribromotrifluoroethane was prepared by first converting CF_3_CH_3_ to CF_3_CHBr_2_ by thermal reaction with Br_2_ in a hot-tube apparatus [6]. Then, in a one-liter, three-necked flask equipped with an efficient stirrer and a reflux condenser, a mixture of 242 g (1 mole) of CF_3_CHBr_2_, 135 g of KOBr (prepared from 112 g of KOH and 160 g of Br_2_), and 300 ml of water was cooled for 3 hr in a bath maintained at 15 °C. The mixture was stirred vigorously and irradiated with a 350-watt bulb throughout the reaction. When the exothermic reaction had subsided and the contents of the flask had cooled, the product solidified. The aqueous layer was decanted. The slightly yellow product, 1,1,1-tribromo-2,2,2-trifluoroethane, was briefly dried in air and sublimed. The yield was 257 g (80%).

## 3. Experimental Procedures

**Figure 1:**
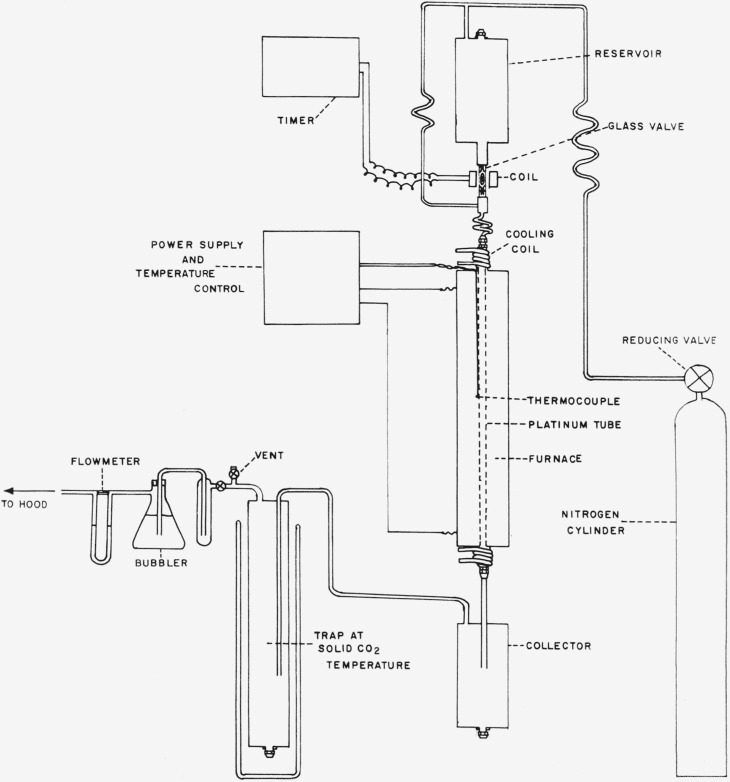
Apparatus for pyrolysis of bromofluorocarbon liquids under pressure.

The pyrolysis experiments were carried out as described previously [2]. However, the apparatus used was newly constructed and designed to handle 2 liters of reactants or about 5 kg of tribromofluoromethane. A diagram of the apparatus is shown in figure 1. The pyrolysis tube, platimun 89 percent and ruthenium 11 percent, 76 cm long, 0.95 cm ID, and 1.15 cm OD, was silver-soldered to brass fittings at each end. The reservoir and traps were constructed of welded stainless steel and connected via copper tubing and brass fittings. The only nonmetal component of the apparatus was a thick-walled, hard-glass, solenoid valve which controlled the input of liquid CBr_3_F mixtures into the furnace. This glass valve was connected with two glass inner 10/30 standard tapers to two outer 10/30 tapers machined out of brass and silver soldered to copper tubing. The taper joints were waxed together with poly (chlorotrifluoroethylene) wax.

The reservoir was filled with the reactants. Then, after closing the system and bringing the furnace to the desired temperature, set on an indicating, proportioning controller operating from a thermocouple, the pressure of N_2_ gas in the system was adjusted, using the reducing valve on the gas cylinder. The flow of N_2_, which had been prepurified, was adjusted by bleeding through a brass, blunt-needle valve at the exhaust end of the apparatus. Finally an electric timer was switched on, which periodically allowed pulses of liquid CBr_3_F mixtures to enter the furnace. After the reservoir was emptied the apparatus was shut down and the pressure allowed to drop to atmospheric. The products were drained from the first trap, which was operated at room temperature. Very little material was found in the second trap, which was operated at −78 °C, but its use tended to prevent corrosion of the exhaust valve. The products were worked up as described previously [1–4].

## 4. Results

Mixtures of CBr_3_F with CBr_4_, with CHBr_3_, with Br_2_, with CBr_2_F_2_, and with CF_3_CBr_3_ were pyrolyzed under conditions listed in table 1. The last three columns in the table give the total amount of recovered material, the weight percent of debromination, and the total weight of products other than bromine. The conditions used were approximately the optimum ones for the production of C_6_F_6_. The experiments with the first three substances listed were carried out to explore the possibility of synthesizing greater amounts of C_6_BrF_5_ and C_6_Br_2_F_4_. After pyrolysis, the material was first treated to remove Br_2_ and then distilled. The fractions collected were analyzed, using a mass spectrometer.

Results from the copyrolysis of CBr_3_F with CBr_4_ are given in table 2, along with boiling points, weights, and analyses of the fractions. Quantitative results, when given, are expressed in mole percent. A few compounds are merely listed when found in trace amounts. The addition of CBr_4_ decreases the yield of C_6_F_6_ and increases the yields of C_6_BrF_5_ and C_6_Br_2_F_4_. However, the major material was the C_6_Br_2_F_4_ and not the more desirable C_6_BrF_5_.

Table 3 gives the results from copyrolysis of the CBr_3_F with CHBr_3_. Surprising amounts of C_6_BrF_5_ and C_6_Br_2_F_4_ are produced. From CHBr_3_ it was initially conceivable that C_6_HF_5_ or even C_6_H_2_F_4_ could be a product. However, no products containing hydrogen were detected, excluding the HBr which was qualitatively evident in the exhaust gases.

Br_2_ seemed to lower the extent of reaction; CBr_2_F_2_ appeared to have no significant effect. These materials are products from the pyrolysis of CBr_3_F itself.

Copyrolysis with CF_3_CBr_3_ gave a significant yield of C_6_F_5_CF_3_, as anticipated (see table 4). This material was also found in the pyrolysis products along with C_6_F_6_ [3] at 800 °C. However, under the conditions used here it is evident that it is produced as a result of the copyrolysis. This material is difficult to separate from the mixture in which it is produced. Unfortunately, our supply of CF_3_CBr_3_ was quite limited, and experiments with greater ratios of this material to CFBr_3_ were not carried out. It is evident, however, that greater ratios would produce such substances as perfluoroxylenes.

## 5. Discussion

The results obtained here are compatible with the type of mechanism assumed previously for this pyrolysis. The stages of reaction can be visualized thus:

**Figure:**
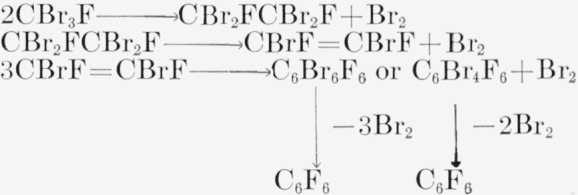


No perhalocyclohexanes have been isolated. The effect of pressure in enhancing the yield can be explained on this type of mechanism [2] without CF ≡ CF as an intermediate. The intermediate, CBrF=CBrF, was found in the products in trace amounts, but no evidence was found for CF ≡ CF as an intermediate. The C_6_Br_6_F_6_ or the C_6_Br_4_F_6_ shown are presumed to be extremely unstable; a Fischer-Hirshfelder model of these compounds indicates large steric effects. No evidence exists for the formation of such compounds from C_6_F_6_ and Br_2_, although the C_6_Cl_6_F_6_ has been made [1]. The chlorine derivative thermally decomposes near 250 to 300 °C.

It has been reported that CF ≡ CF forms a dimer, presumably tetrafluorocyclobutadiene [7]. So far, no evidence exists for these compounds from the pyrolysis reaction of CBr_3_F. However, CF ≡ CH does trimerize to 1,2,4-trifluorobenzene [8], and hexafluoro-2-butyne trimerizes to hexa(trifluoromethyl)benzene [9].

## Figures and Tables

**Table 1:** Pyrolysis of bromofluorocarbon mixtures

Mixtures	Weight	Moles	Temperature	Pressure	N_2_ flow	Time	Recovered material (including Br_2_)	Br_2_ removal	Products (other than Br_2_)

	*g*		*°C*	*atm*	*cm*^3^*/min*	*hr*	%	%	*g*
CBr_3_F CBr_4_	2981 756	11 2.2	} 540	18	25	5	98	87	652
CBr_3_F CBr_4_	7859 1307	29 3.8	} 540	18	25	7	67	87	1721
CBr_3_F CBr_4_	4490 1100	16.5 3.3	} 545	3.5	25	5.2	98.5	69.8	740
CBr_3_F CHBr_3_	4065 759	15 3.1	} 560	10	25	11.3	95	96	769
CBr_3_F Br_2_	3400 1360	12.5 8.5	} 540	10	50	11	70	49^a^	416 9C_6_F_6_ 197CBr_3_F 210Residue
CBr_3_F CBr_2_F_2_	2710 420	10 2	} 540	10	25	5	77	68	420 84C_6_F_6_ 210CBr_3_F 126Residue
CBr_3_F CF_3_CBr_3_	1900 210	10.7 1	} 560	10	25	4	98	74	793

a Does not include the added bromine.

**Table 2:** Fractional distillation of products from the pyrolysis of CBr_3_F/CBr_4_ mixtures

Boiling range	Pressure	Weight of fraction	Analysis of fraction

°*C*	mm	g	*Mole* %
25 to 90	at 760	139	C_6_F_6_ (80%), CBrFCBrF (10%), CBr_2_F_2_
90 to 120	at 760	848	CBr_3_F (90%), C_3_Br_4_F_2_, C_6_BrF_5_
120 to 145	at 760	160	C_6_BrF_5_ (79%), C_2_Br_4_F_2_ (20%), C_7_BrF_7_
85 to 95	at 25	218	C_6_Br_2_F_4_ (90%), C_2_Br_4_F_2_, C_6_BrF_5_
88 to 98	at 10	61.5	C_6_Br_2_F_4_ (50%), C_2_Br_4_ (10%)
>100	at 10	268	Not analyzed.

**Table 3:** Fractional distillation of products from the pyrolysis of CBr_3_F/CHBr_3_ mixtures

Boiling range at 760 mm	Weight of fraction	Analysis of fraction

*°C*	*g*	*Mole* %
45 to 100	69.4	C_6_F_6_ (70%), CHFBr_2_ (20%)
100 to 125	510.4	CBr_3_F (90%), C_2_Br_3_F
125 to 140	69.1	C_6_BrF_5_ (60%), CHBr_3_ (30%), C_7_BrF_7_, C_6_HBrF_4_
140 to 175	39.7	C_4_Br_3_F_3_, C_3_Br_3_F_3_, C_8_BrF_7_
175 to 200	94.8	C_6_Br_2_F_4_, C_2_Br_4_F_2_

**Table 4:** Fractional distillation of products from the pyrolysis of CBr_3_F/CF_3_CBr_3_ mixtures

Boiling range at 760 mm	Weight of fraction	Analysis of fraction

*°C*	*g*	*Mole* %
50 to 95	51.2	C_6_F_6_ (66%), C_6_F_5_CF_3_ (5%), C_6_F_4_Br_2_
95 to 106	51.7	CBr_3_F (85%), C_7_F_8_ (10%), C_6_F_6_ (5%)
106 to 110	302.9	CBr_3_F (90%), C_7_F_8_ (5%), C_2_Br_3_F_3_
>110	401	Not analyzed.

## Footnotes

1 Figures in brackets indicate the literature references at the end of this paper.

2 The courtesy of the General Chemical Division, Allied Chemical and Dye Corporation, in supplying this material is gratefully acknowledged.
